# Oral symptom assessment tools in patients with advanced cancer: a scoping review

**DOI:** 10.1007/s00520-022-07169-1

**Published:** 2022-06-03

**Authors:** Niamh Cleary, Olivia Munnelly Mulkerrin, Andrew Davies

**Affiliations:** 1 Trinity College Dublin, Dublin, Ireland; 2 Our Lady of Lourdes Hospital, Drogheda, Drogheda, Ireland; 3 Our Lady’s Hospice Dublin, Dublin, Ireland; 4 University College Dublin, Dublin, Ireland

**Keywords:** Oral care, Oral health, Neoplasms, Palliative care, Symptom assessment

## Abstract

**Purpose:**

Oral symptoms are common in patients with advanced cancer. The aim of this scoping review was to identify oral symptom assessment tools that have been specifically utilised in patients with “advanced cancer”.

**Methods:**

The review was conducted/reported according to international guidelines for undertaking scoping reviews. PubMed, Embase, and CINAHL were searched for articles involving adult patients with advanced cancer, which involved assessment of ≥ 2 oral symptoms, and which involved patients with > 1 type of cancer.

**Results:**

The review identified four validated symptom assessment scales, including one cancer-specific quality of life scale (EORTC QLQ OH-15), one generic tool for assessing the “social impact” of specific oral problems (OHIP), one cancer-specific generic symptom assessment scale (MSAS), and one cancer-specific oral symptom assessment scale (OSAS).

**Conclusion:**

Symptom assessment tools can facilitate good symptom control in clinical practice, and are an integral component of clinical research. The review identified four validated symptom assessment scales that could be utilised to assess oral symptoms in patients with advanced cancer.

**Supplementary Information:**

The online version contains supplementary material available at 10.1007/s00520-022-07169-1.

## Introduction

One of the main aspects of palliative care is the management of “pain and other distressing symptoms” [1]. Patients with advanced cancer experience a range of different symptoms, including a variety of different oral symptoms [2]. Thus, Davies et al. [3] reported that 97.5% of participants in their multicentre study experienced at least one oral symptom, and that the median number of oral symptoms experienced was five (range 1–18). Moreover, many of these oral symptoms had a high frequency and a high intensity, and were associated with significant distress/ “bothersomeness” (and negative impact on quality of life). For example, 79.6% of participants experienced xerostomia/dry mouth, and this was the third most common symptom overall (after “lack of energy” and “feeling drowsy”) [3].

Investigators have identified discrepancies between the recorded prevalence of oral symptoms and the true (higher) prevalence of these symptoms in patients with advanced cancer [4]. The reasons for the latter are unclear. Healthcare professionals may not enquire about a symptom if (a) they perceive the symptom to be uncommon; (b) they perceive the symptom to be unimportant; (c) they perceive there is no treatment for the symptom; and/or (d) time does not permit. Similarly, patients may not volunteer a symptom if (a) they perceive the symptom to be inevitable; (b) they perceive there is no treatment for the symptom; (c) they sense that healthcare professionals perceive the symptom to be unimportant; and/or (d) other symptoms predominate.

Symptom assessment tools can facilitate good clinical practice by improving the thoroughness of the assessment (and re-assessment) of common symptoms. Furthermore, validated symptom assessment tools are essential to undertaking robust research studies. However, many generic symptom assessment scales contain no oral symptoms (e.g., Edmonton Symptom Assessment Scale/ESAS [5]), or only a limited number of oral symptoms (e.g., Memorial Symptom Assessment Scale/MSAS [6]). For example, the MSAS, which consists of 32 symptoms (26 physical, 6 psychological), includes only four oral symptoms, i.e. “dry mouth”, “change in the way food tastes”, “difficulty swallowing”, and “mouth sores”. Interestingly, the Norwegian version of ESAS does include xerostomia, as this is one of the “10 common symptoms of advanced cancer” [7].

The aim of this scoping review was to identify/describe oral symptom assessment tools that have been specifically utilised in patients with “advanced cancer” [8], and particularly in cancer patients receiving symptom-oriented treatment (i.e. palliative care).

## Methods

The function of a scoping review is to identify the available evidence rather than to produce critically appraised answers to research questions [9, 10]. The Arksey and O’Malley methodological framework [11], which has been enhanced/developed by Levac et al. [12] and the Joanna Briggs Institute [13], was used as a framework to conduct this scoping review. The PRISMA Extension for Scoping Reviews (PRISMA-ScR) checklist was used as a guide in reporting this scoping review [14].

### Study eligibility criteria

We used the Population, Intervention, Comparator/control, Outcome and Study design (PICOS) framework to identify relevant research studies [15]. Eligible studies included adult patients with advanced cancer (as defined by the National Cancer Institute/NCI, USA): “cancer that is unlikely to be cured or controlled with treatment” [8]. Studies involving patient-rated oral symptom assessment tools, as well as quality of life (QoL) instruments that contain oral symptom items, were included. However, studies involving assessment of a single oral symptom (e.g., xerostomia), or a single cancer site (e.g., head and neck cancer), were not included. Observational and experimental studies were included. Perspective, commentary or opinion articles without original data were excluded.

### Search strategy

A comprehensive search of three electronic databases including PubMed, Embase, and CINAHL was conducted from inception to 11 June 2021. The literature review was adapted to meet the requirements of each database searched with guidance from a health sciences librarian. The search was limited to English language articles. Reference lists of all eligible full texts were hand searched for other relevant articles. In addition, handsearching of reference lists of relevant chapters in academic textbooks was undertaken to ensure a comprehensive search of the literature was conducted.

### Data management and synthesis

The EndNote 20™ (Clarivate) bibliographic software was used to store the records retrieved from all the literature searches. This software enables duplicates to be removed. We then used the Covidence software to screen these records. Two reviewers (NC, OM) independently screened the titles and abstracts using the PICOS criteria. Where an abstract was unavailable, the paper was included in the full text review process. If there was any conflict between the two reviewers, a third reviewer was available to determine inclusion. The same two reviewers independently reviewed the full texts, and extracted the relevant information using a review-specific proforma. Again, if there was any conflict between the two reviewers, a third reviewer was available to determine inclusion.

## Results

### Search results

**Fig. 1:**
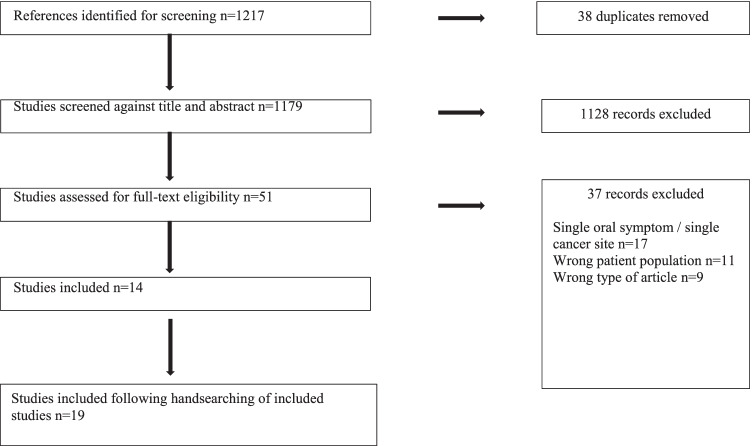
Study flow

The search strategy identified 1179 unique references (Fig. 1). Fourteen papers were included in the full data extraction. One reference was a conference abstract and the authors confirmed that this was the same study as an included article. Five further articles were included following handsearching of the included full text articles. No further articles were included following handsearching of relevant chapters in academic textbooks.

### Symptoms assessed

The number of oral symptoms assessed in each study varied from two [22, 23] to 20 [3, 36]. Xerostomia (“dry mouth”) was universally assessed, which is unsurprising as studies in patients with advanced cancer demonstrate a very high prevalence (i.e. 82–83.5%) [3, 36, 37]. Other commonly assessed symptoms included oral discomfort (18/18 studies), taste disturbance (15/18 studies), and denture problems (8/18 studies) (see Table 1).

**Table 1:** Summary of data extraction

Reference	Study type	Number of oral symptoms assessed	Symptoms assessed	Symptom dimensions assessed	Time frame	Symptom assessment tool
Pople et al., 1986 [16]	Observational	3	“Dry mouth”; “altered taste”; “sore mouth”	Present/absent	Not described (but assessments performed on day 1, day 3, and day 5 after admission)	Non validated questionnaire
Clarke et al., 1987 [17]	Observational	5	“Xerostomia”; “glosso-dynia”; “painful gums”; “dysphagia”; “sour taste”	Present/absent	Not described	Non validated questionnaire
Aldred et al., 1991 [18]	Observational	6	“Disturbance of taste”; “dysphagia”; “oral soreness”; “dryness of the mouth”; “difficulty in wearing dentures”; “any other miscellan-eous problems”	Present/absent	Not described	Non validated questionnaire
Jobbins et al., 1992 [19]	Observational	5	“Xerostomia”; “denture problems”; “taste disturbance”; “swallowing difficulty”; “oral soreness”	Present/absent	Not described	Non validated questionnaire
Sweeney et al., 1997 [20]	Interventional – clinical trial in patients with xerostomia	7	“Oral dryness during the day”; “dryness at night”; “soreness of the mouth”; “unpleasant or altered taste sensation”; “difficulty talking”; “difficulty eating”; “denture problems”	Present/absent Visual analogue scales used to assess response (no details provided)	Not described	Non validated questionnaire
Sweeney et al.,1998 [21]	Observational	7	“Dry mouth during the day”; “dry mouth at night”; “soreness of the mouth”; “bad or altered taste”; “difficulty talking”; “difficulty eating”; “problems with dentures”	Present/absent “Grading”—visual analogue scale 0–6 (no problem-severe problem)	Not described	Non validated questionnaire
Oneschuk et al., 2020 [22]	Observational	2	“Mouth pain”; “mouth dryness”	Presence/absence Intensity—numerical rating scale 0–10 (low–high) “Importance” (relative to other symptoms/problems)—not important, slight importance, some importance, moderate importance, considerable importance, very important, great importance	Not described (but symptoms reported present from 1 day to > 1 year)	Non validated questionnaire
Milligan et al., 2001 [23]	Interventional – oral care regimen	2	“Oral pain”; “oral dryness”	Presence/absence	Not described (but assessments performed on day 1, and day 7)	Non validated questionnaire
Davies et al., 2001 [24]	Observational	4 (“standard” symptoms) 3 (“additional” symptoms)	“Dry mouth”; “difficulty swallow-ing”; “mouth sores”; “change in the way food tastes” “Oral discomfort”; “difficulty chewing”; “difficulty speaking”	Presence/absence Frequency—rarely, occasionally, frequently, almost constantly Intensity—slight, moderate, severe, very severe Amount of distress caused—not at all, a little bit, somewhat, quite a bit, very much	Previous week	Validated questionnaire (with additional questions)—Memorial Symptom Assessment Scale/MSAS [6]
Alt-Epping et al., 2012 [25]	Observational	5	“Dry mouth”; “taste disturbances”; “dysphagia”; “halitosis”; “pain of the oral cavity”	Presence/absence Intensity—numerical rating scale 0–10 (low–high)	Not described	Non validated questionnaire
Wilberg et al., 2012 [7]	Observational	5	“Oral discomfort”; “xerostomia”; “taste disturbances”; “problems eating”; “dental health”	Presence/absence	Not described (but xerostomia reported present for > 3 months)	Non validated questionnaire
Hjermstad et al., 2012 [26]	Observational	14	“Pain in gums”; “bleeding gums”; “lip sores”; “problems with teeth”; “sore mouth”; “sores in mouth corners”; “dry mouth”; “sticky saliva”; “sensitive mouth”; “taste change”; “problems with solid food”; “trouble enjoying meals”; “worn dentures”; “ill-fitting dentures”	Presence/absence “Extent to which you have experienced these symptoms”—not at all, a little, quite a bit, very much	Previous week	Development study—EORTC QLQ-OH17
Fischer et al., 2014 [27]	Observational	3 main symptoms (with extra questions about related oral symptoms)	“Xerostomia” (with extra question about dry lips); “orofacial pain” (separate questions about intraoral and facial pain, with extra question about mouth sores); “taste change”	Presence/absence Frequency—Likert scale 0–4 (never-always) Severity (xerostomia, orofacial pain)—numerical rating scale 0–10 (low–high)	Not described	Non validated questionnaire (Oral Problem Scale)—based upon questions from other tools, including Oral Health Impact Profile/OHIP [28], and Oral Symptom and Function Scale [29]
Mercadante et al., 2015 [30]	Observational	3	“Limitation on nutrition or hydration” (due to mucositis); “dry mouth”; “dysphagia”	Presence/absence Mucositis intensity—no limitation, mucositis partially preventing nutrition or hydration, mucositis severely limiting nutrition or hydration, mucositis completely preventing nutrition or hydration Dry mouth/dysphagia intensity—numerical rating scale 0–10 (low–high)	Not described	Non validated questionnaire
Nikles et al., 2015 [31]	Interventional – clinical trial in patients with xerostomia	3 generic symptoms 4 symptoms from Xerostomia Inventory (see final column) ? number symptoms from OHIP-49 (see final column)	“Dry mouth”; “difficulty in swallowing”; “altered taste” “Mouth feels dry”; “difficulty eating dry food”; difficulties swallowing certain foods; “lips feel dry” Symptoms different for different versions	Presence/absence Severity—numerical rating scale 0–10 (low–high), and summated score from Xerostomia Inventory (see below) Frequency—never. hardly ever, occasionally, fairly often, very often Frequency—scales different for different versions (see final column)	Previous 24 h Not described Not described	Non validated questionnaire—patients were also asked to complete Xerostomia Inventory/XI [32], and OHIP [28]. The XI is a validated single symptom assessment tool. The OHIP is a validated assessment tool that measures “the social impact of oral disorders”. The protocol paper states that the OHIP-14 (14 questions) would be used [33] but the main paper suggests that the OHIP-49 (49 questions) was used [31]
Hjermstad et al., 2016 [34]	Observational	12	“Pain in your gums”; “problems with bleeding gums”; “lip sores”; “problems with your teeth”; “soreness in your mouth”; “sores in the corners of your mouth”; “dry mouth”; “sticky saliva”; “mouth been sensitive to food and drink”; “food and drink tasted different than usual”; “problems eating solid foods”; “problems with an ill-fitting denture”	Presence/absence “Extent to which you have experienced these symptoms”—not at all, a little, quite a bit, very much	Previous week	Validation study—EORTC QLQ-OH17 (leading to EORTC QLQ-OH15)
Magnani et al., 2019 [35]	Interventional – oral care regimen	3	“Xerostomia/dry mouth”; “dysgeusia”; “orofacial pain”	Presence/absence Intensity—numerical rating scale 0–10 (low–high)	Not described (but assessments performed on day 1, and day 3)	Non validated questionnaire
Davies et al., 2021 [3, 36]	Observational	20 (with option to report additional oral symptoms)	“Dry mouth”; “mouth discomfort/pain”; “lip discomfort”; “cracking of lips”; “cracking of corner of mouth”; “taste disturbance”; “difficulty chewing”; “difficulty swallow-ing”; “difficulty speaking”; “‘dirty’ mouth”; “coating of tongue”; “bad breath”; “toothache/pain in teeth”; “sensitivity of teeth”; “jagged teeth”; “denture fitting problems”; “bleeding from mouth”; “burning sensation in mouth”; “altered sensation in mouth”; “ulcers in mouth”	Presence/absence Frequency—rarely, occasionally, frequently, almost constantly Intensity—slight, moderate, severe, very severe Amount of distress caused—not at all, a little bit, somewhat, quite a bit, very much	Previous week	Oral Symptom Assessment Scale/OSAS Initial validation of OSAS undertaken as part of this study

It should be noted that the wording of the questions differed amongst the oral assessment tools: for example, taste disturbance was variously described as “altered taste”, “sour taste”, “disturbance of taste”, “taste disturbance(s)”, “unpleasant or altered taste sensation”, “bad or altered taste”, “change in the way food tastes”, “taste change”, “food and drink tasted different from usual”, and “dysgeusia” (although unclear as to the term used with the participants) [35].

Davies et al. [3] assessed 20 oral symptoms, many of which were not included in other studies (e.g. “sensitivity of teeth”, “altered sensation in mouth”, “burning sensation in mouth”, “bleeding from mouth”), and all of which were present in $\ge 7.5\%$ of participants. Moreover, Davies et al. [3] identified a number of so-called oral symptom “clusters” in this cohort of patients, i.e. symptoms that frequently co-existed.

### Dimensions assessed

Many of the (generally older) studies simply assessed the presence or absence of specific oral symptoms [7, 16–19, 23]. However, many of the (generally newer) studies assessed one or more dimensions, including frequency [3, 24, 27, 31, 36], intensity/severity [3, 21, 22, 24, 25, 27, 30, 31, 35, 36], level of distress or bothersomeness [3, 24, 36], level of limitation [30], and relative importance [22].

### Symptom time frames

Many of the studies did not specify the time frames used in the questions. Of the studies that did specify a time frame, this varied from “in the previous 24 h” [31], to “during the past week” [3, 26, 34, 36]. Nevertheless, some studies appear to have used longer time frames based upon the results reported (e.g., up to 1 year [22]).

## Discussion

As highlighted, oral symptoms are common in patients with advanced cancer. Furthermore, these symptoms are often frequent in occurrence, moderate to severe in intensity, and cause significant levels of distress (and so have a negative impact on quality of life). However, observational studies suggest that oral problems are not well-managed in this group of patients [38]. The reasons for the latter are several, and include inadequate assessment (including non-identification of oral symptoms/problems), inappropriate treatment, and inadequate re-assessment.

Symptom assessment tools can improve clinical practice through the improved/earlier identification of troublesome “orphan” symptoms (i.e. symptoms not usually reported or assessed) [39]. The “ideal” symptom assessment tool should be valid, reliable, relevant (for the population/specific scenario), comprehensive (for the specific scenario), multidimensional, and easy to administer/complete [40]. Symptom assessment tools also have a role in research, in both observational studies, and in interventional studies (as a means of demonstrating improvements in symptom control).

Currently, there is no consensus on the number of symptoms that should be included in symptom assessment tools. Longer (more comprehensive) symptom assessment tools may be more suited to research settings, whilst shorter assessment tools may be preferable for clinical practice due to related issues of patient burden, and inadequate completion.

Symptom assessment tools that only ask about the presence (or absence) of a symptom, or are limited to the assessment of a single dimension (e.g. frequency), risk under-estimating, and equally over-estimating, the importance of certain symptoms. For example, although a symptom may be frequent in nature, it may not cause significant distress (and so may not require any intervention). It should be noted that the level of distress of a symptom is often a very good indicator of its impact on the person’s quality of life (although frequency, and especially intensity/severity, is also important) [40].

Many of the included studies used study-specific questionnaires, which had not been validated, although some contained elements from other validated assessment tools (see Table 1). Validated tools included the MSAS [24], the EORTC QLQ-OH17 [26], the EORTC QLQ-OH15 [34], and the OSAS [3, 36]. One study used the OHIP [31], although related results were not presented (and it was unclear which version was used). Of note, another study used selected elements from the OHIP [27].

The MSAS is a 32-item multidimensional generic symptom assessment scale, which has been extensively validated in cancer patients [6]. It contains four oral symptoms (i.e. “dry mouth”, “difficulty swallowing”, “mouth sores” and “change in the way food tastes”). The MSAS also provides blank spaces for the patient to add additional symptoms not mentioned within the tool. Davies et al. [24] supplemented the MSAS with three further oral symptoms (“oral discomfort”, “difficulty chewing” and “difficulty speaking”). The MSAS involves patients rating the frequency, severity, and distress caused by each of the physical symptoms.

The EORTC QLQ oral health module is a validated quality of life instrument, which includes a number of oral symptoms. It was initially developed as the EORTC QLQ OH-17 (a 17-item tool) [26], but was subsequently refined to the EORTC QLQ-15 (a 15-item tool) [34]. This oral health module must be completed alongside the core EORTC QLQ C-30 instrument. The EORTC QLQ-15 assesses 12 oral symptoms with three further items pertaining to the wearing of dentures, and information received about dental or mouth problems.

The OSAS is a novel 20-item multidimensional oral symptom assessment tool, which has undergone initial validation in patients with advanced cancer (and is currently undergoing further validation in this group of patients) [3, 36]. The OSAS was modelled on the MSAS. The symptoms assessed are shown in Table 1, and it also provides blank spaces for the patient to add any additional oral symptoms not mentioned within the tool. The OSAS involves patients rating the frequency, severity, and distress caused by each of the oral symptoms.

## Conclusion

Symptom assessment tools can facilitate good symptom control in clinical practice, and are an integral component of clinical research.This scoping review identified four validated symptom assessment scales that could be utilised to assess oral symptoms in patients with advanced cancer, including one cancer-specific quality of life scale (EORTC QLQ OH-15), one generic tool for assessing the “social impact” of specific oral problems (OHIP), one cancer-specific generic symptom assessment scale (MSAS), and one cancer-specific oral symptom assessment scale (OSAS).

## Supplementary Information

Below is the link to the electronic supplementary material.

Supplementary file1 (DOCX 20 KB)
