# Supplementary material for: Oral symptom assessment tools in patients with advanced cancer: a scoping review
Source: Support Care Cancer. 2022 Jun 3;30(9):7481–90. doi: 10.1007/s00520-022-07169-1 (PMC9385820; doi:10.1007/s00520-022-07169-1)
Supplement: Supplementary file 1 — Supplementary file1 (DOCX 20 KB) [file 520_2022_7169_MOESM1_ESM.docx]

PubMed

(((((((((((((((oral care[Title/Abstract]) OR (oral symptom*[Title/Abstract])) OR ("oral health related quality of life"[Title/Abstract])) OR (oral disease*[Title/Abstract])) OR (mouth inflammation[Title/Abstract])) OR (dental disease[Title/Abstract])) OR (dental health[Title/Abstract])) OR (oral manifestations[MeSH Terms])) OR (oral health[MeSH Terms])) OR (oral candidiasis[MeSH Terms])) OR (mouth diseases[MeSH Terms])) OR (oral hygiene[MeSH Terms])) OR (stomatitis[MeSH Terms])) OR (xerostomia[MeSH Terms]))

AND

((((((((((health impact assessment[MeSH Terms]) OR (patient reported outcome measures[MeSH Terms])) OR (patient outcome assessment[MeSH Terms])) OR (problem rating scale for outcomes[Title/Abstract])) OR (outcome measure*[Title/Abstract])) OR (outcome assessment*[Title/Abstract])) OR (outcome tool*[Title/Abstract]))) OR (symptom assessment scale*[Title/Abstract])) OR (clinical assessment tool*[Title/Abstract])))

AND

((((((((((advanced cancer[Title/Abstract]) OR (metastatic cancer[Title/Abstract])) OR (terminal cancer[Title/Abstract])) OR (care, palliative[MeSH Terms])) OR (medicine, palliative[MeSH Terms])) OR (end of life care[Title/Abstract])) OR (Neoplasms[MeSH Terms])) OR (neoplasm metastasis[MeSH Terms])) OR (neoplasm metastases[MeSH Terms])) OR

(terminal care[Title/Abstract]))

# CINAHL

patient outcome assessment* OR problem rating scale for outcome* OR outcome measure* OR outcome tool* OR outcome measurement tool* OR symptom assessment scale* OR MH outcome assessment OR MH clinical assessment tools OR MH patient assessment OR MH patient-reported outcomes OR MH health impact assessment

AND

MH palliative care OR MH palliative medicine OR MH Neoplasms OR MH terminal care OR MH Neoplasm metastasis OR advanced cancer OR terminal cancer OR metastatic cancer OR terminal care OR end of life care

AND

MH oral manifestations OR MH oral health OR MH oral candidiasis OR MH mouth diseases OR MH oral hygiene OR MH xerostomia OR MH stomatitis OR oral care OR oral symptom* OR "oral health related quality of life" OR oral disease OR dental disease OR dental health

Embase

health impact assessment or patient reported outcome measures or patient outcome assessment or clinical assessment tool* or "problem rating scale for outcomes" or outcome measure* or outcome assessment* or outcome tool* or outcome measurement tool* or symptom assessment scale*).mp. [mp=title, abstract, heading word, drug trade name, original title, device manufacturer, drug manufacturer, device trade name, keyword, floating subheading word, candidate term word]

AND

(oral care or oral symptom* or "oral health related quality of life" or oral disease or mouth inflammation or dental disease or dental health or oral manifestations* or oral health or oral candidiasis or mouth disease* or oral hygiene or stomatitis or xerostomia).mp. [mp=title, abstract, heading word, drug trade name, original title, device manufacturer,drug manufacturer, device trade name, keyword, floating subheading word, candidate term word]

|  |
| --- |

AND

(medicine, palliative or care, palliative or Neoplasms or Neoplasm metastasis or end of life care or terminal care or terminal cancer or advanced cancer or metastatic cancer).mp. [mp=title, abstract, heading word, drug trade name, original title, device manufacturer, drug manufacturer, device trade name, keyword, floating subheading word, candidate term word]
